# Detection of the ST111 Global High-Risk *Pseudomonas aeruginosa* Clone in a Subway Underpass

**DOI:** 10.3390/cimb47070532

**Published:** 2025-07-09

**Authors:** Balázs Libisch, Chioma Lilian Ozoaduche, Tibor Keresztény, Anniek Bus, Tommy Van Limbergen, Katalin Posta, Ferenc Olasz

**Affiliations:** 1 Agribiotechnology and Precision Breeding for Food Security National Laboratory, Institute of Genetics and Biotechnology, Hungarian University of Agriculture and Life Sciences, 2100 Gödöllő, Hungary; ozoaduche.chioma.lilian@phd.uni-mate.hu (C.L.O.); kereszteny.tibor@uni-mate.hu (T.K.); posta.katalin@uni-mate.hu (K.P.); olasz.ferenc.gyorgy@uni-mate.hu (F.O.); 2 Doctoral School of Biology, Hungarian University of Agriculture and Life Sciences, 2100 Gödöllő, Hungary; 3 Sustainable Environment Development Initiative (SEDI), Benin City 300102, Nigeria; 4 Anitom BV, B-3800 Sint-Truiden, Belgium; anniek.bus@anitom.be (A.B.); tommy.vanlimbergen@anitom.be (T.V.L.)

**Keywords:** One Health, *P. aeruginosa*, multidrug resistance, serotype O12, serotype O4, sequence type ST111, molecular epidemiology, high-risk clone, clonal dissemination, carbapenemase

## Abstract

*P. aeruginosa* strain NL201 was cultured from an urban water drain in a populated subway underpass as an environmental isolate for the ST111 global high-risk *P. aeruginosa* clone. In addition to carrying generally present intrinsic *P. aeruginosa* antibiotic resistance genes, this serotype O4 isolate also carries a set of additional acquired resistance determinants, including *aadA2*, *bla*_OXA-10_, *sul1*, and an *aac(6′)-Ib* family gene. The NL201 isolate features the *bla*_PDC-3_ allele, which was found to confer significantly higher catalytic efficiency against cefepime and imipenem compared to *bla*_PDC-1_, as well as the potent *P. aeruginosa* virulence factors *exoS, exoT*, and *algD.* Serotype O4 isolates of the ST111 global high-risk *P. aeruginosa* clone have been reported from clinical samples in Canada and the USA, human stool samples in France, and environmental samples (such as cosmetic, hospital drains, and urban water drain) from various European countries. These observations underscore the effective dissemination of the ST111 global high-risk *P. aeruginosa* clone between different hosts, environments, and habitats, and they warrant targeted investigations from a One Health perspective on the possible routes of its spread and molecular evolution.

## 1. Introduction

*Pseudomonas aeruginosa* is a key host Gram-negative bacterium for potent acquired antibiotic resistance genes (ARGs), including acquired metallo-carbapenemases, such as VIM, IMP, SPM, or NDM-type β-lactamases. *P. aeruginosa* has a panmictic/heterogenous population structure, which also contains certain high-risk multidrug-resistant (MDR) clones [1,2]. Carbapenem resistance in this species may be partially conferred by horizontally acquired carbapenemases, but it can also emerge through mutations leading to the loss of the OprD porin (for imipenem resistance) or through a combination of the loss of OprD with upregulated efflux pumps, particularly MexAB-OprM (for meropenem and doripenem resistance) [1].

Molecular typing studies of multidrug-resistant *P. aeruginosa* clinical isolates in the second half of the 2000s identified several high-risk multidrug-resistant (MDR) *P. aeruginosa* clones with the (founder) sequence types ST111, ST235, ST175, and ST395 [3,4,5,6,7,8]. Among these high-risk clones, resistance markers in representative isolates of the ST175 clone in Spain showed that the extensively drug-resistant (XDR) pattern was triggered by the combination of AmpC hyperproduction, OprD inactivation, additional mutations yielding high-level fluoroquinolone resistance (GyrA T83I and D87N, and ParC S87W), MexXY-OprM efflux overexpression, and the carriage of a class 1 integron harboring the *aadB* gene [9]. Similarly, an MDR ST175 *P. aeruginosa* clone showing a countrywide distribution in Hungary displayed AmpC-mediated resistance to ceftazidime, and it harbored a class 1 integron with *aadB* and *aadA13* aminoglycoside adenylyltransferase gene cassettes in its variable region, contributing to phenotypic resistance against gentamicin and tobramycin [7,9]. Isolates of the ST175, ST111, and ST235 epidemic high-risk clones with the MDR or XDR phenotypes showed increased biofilm formation and mutant frequencies, but motility, pyoverdine and pyocyanin production, and fitness were reduced [10].

ST111 was proposed to be the second most widespread high-risk *P. aeruginosa* clone after ST235 in 2020 worldwide. It is strongly associated with MDR/XDR phenotypes and frequently produces a VIM-2 metallo-β-lactamase (MBL), which has been detected in over 20 countries in Europe, Asia, and the Americas in ST111 isolates [11]. Genetic analysis suggested that ST111 originally had serotype O4 but subsequently acquired the determinants for serotype O12 through recombination, together with a quinolone resistance mutation in *gyrA* (*gyrA*^C248T^), which might have also facilitated the dissemination of serotype O12 in clinical settings [12]. The *gyrA* C248T mutation leading to a Thr83Ile amino acid substitution is also one of the most common mutations detected in ciprofloxacin-resistant *P. aeruginosa* isolates [13].

MDR ST111 isolates from hospitals in Bulgaria displayed a combination of acquired β-lactamase production (VEB-1 and OXA-types) coupled with the lack of OprD porin and the overexpression of *mexXY-oprM*, which resulted in a carbapenem-resistant phenotype without harboring any acquired MBL genes [14]. Various oxacillinases, such as *bla*_OXA-2_, *bla*_OXA-9_, *bla*_OXA-10_, and others, were also reported from ST111 isolates, together with *aac(6)-Ib* and *aacA29* determinants of aminoglycoside resistance. Amino acid substitutions in GyrA, ParC, and/or ParE were also confirmed in fluoroquinolone-resistant ST111 *P. aeruginosa* isolates [15]. The dissemination of MDR *P. aeruginosa* is considered to be clonal, and the carriage of GyrA T83I and ParC S87L double mutations in *P. aeruginosa* was observed in all three major international ST111, ST235, and ST175 MDR clones [16]. Although MDR strains of *P. aeruginosa* often display attenuated virulence compared to susceptible isolates, several strains of the high-risk clones ST111, ST235, and ST773 have been observed to show considerable virulence that could be linked to the production of the exoU toxin [10,16]. Epidemic MDR *P. aeruginosa* clones have also been associated with poorer patient prognoses [17].

Among *P. aeruginosa* isolates collected from canine otitis externa cases in different geographic locations in Europe, the identification of the ST111 sequence type indicates the potential for its dissemination between humans and dogs, thus posing a risk of zoonotic spread, particularly to immunocompromised individuals [18]. A carbapenem-resistant ST111 *P. aeruginosa* isolate from Guanay Cormorants from Isla Pescadores, Lima, Peru, carried both *bla*_VIM_ (encoded within a class 1 integron) and *bla*_IMP_. The isolate also harbored *exoS* and *exoY* but neither *exoU* nor *algD.* The authors noted that the droppings (guano) of Guanay Cormorants and other birds are widely used on local rural farms as organic fertilizers; therefore, the dissemination of associated ST111 carbapenem-resistant *P. aeruginosa* isolates into agriculture is also possible in this way [19]. ST111 *P. aeruginosa* has also been isolated from sick ruminant livestock in Saudi Arabia [20] and water samples collected in the Campania Region of Southern Italy [21].

A phylogenetic analysis on a global collection (*n* = 969) of ST111 *P. aeruginosa* isolates revealed three clades (A, B, C) and two subclades (C1, C2) [2]. Clade ST111-A was identified as the ancestral clade, while clades B, C1 and C2 likely emerged during the 1700s and 1800s. Subclade ST111-C2 currently dominates the global ST111 *P. aeruginosa* population. All the clade A and B strains and >80% of subclade ST111-C1 had O4 serotype. In contrast, all but two subclade C2 isolates possessed O12 serotype. Subclade ST111-C2 (currently the most dominant cluster) was proposed to have evolved from ST111-C1 in the second half of the 1800s, followed by the establishment of *bla*_VIM_ containing integrons among ST111-C2 strains from the 1960s onwards [2].

The first known VIM-producing serotype O12 *P. aeruginosa* clinical isolates of the ST111 clonal complex were identified in Hungary between 2002 and 2005, originating from clinical settings in Budapest and Pécs [5,22,23,24]. Based on a detailed analysis of integron structures carrying *bla*_VIM_ cassettes and the in vitro susceptibility profiles of serotype O1 ST313 *P. aeruginosa* clinical isolates, the presence of a local environmental ARG pool was proposed. This may have interacted with, and contributed to, ARG exchanges with the acquired resistome of clinical settings as well [5].

Considering these earlier observations and the proposed existence of a local environmental ARG pool, the main objectives of the current study were as follows: (1) to perform a pilot, targeted sampling of selected urban environmental sites to detect MDR *P. aeruginosa*, and (2) to characterize the potentially isolated MDR environmental *P. aeruginosa* strains using genomics methods and to compare them with *P. aeruginosa* isolates of clinical origin. During this work, a serotype O4 ST111 environmental *P. aeruginosa* strain was isolated in Budapest from a subway underpass located in the neighborhood of several healthcare institutions.

## 2. Materials and Methods

### 2.1. Isolation and Identification of P. aeruginosa Strain NL201

**Figure 1:**
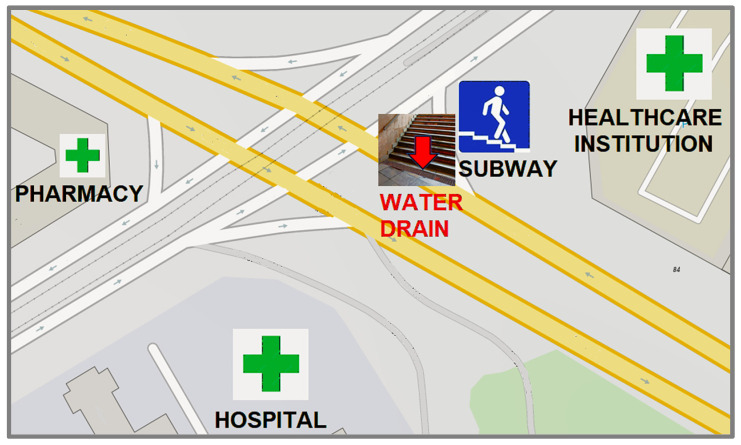
The urban environment where *P. aeruginosa* strain NL201 was isolated from a wet water drain in a subway underpass at a major roads crossing. The location of sampling is indicated by a red arrow. Small grey arrows indicate the direction of traffic.

*P. aeruginosa* strain NL201 was isolated in October 2023 from an urban water drain sample collected in a subway underpass in Budapest (Figure 1). Sterile Cary-Blair specimen collection swabs were used for sampling, which were subsequently placed in a sterile tube containing transport medium (Deltalab, Barcelona, Spain). The swabs were then streaked on violet red bile glucose (VRBG) agar plates (VWR International, Leuven, Belgium) containing 8 mg/L ampicillin + 8 mg/L tetracycline + 2 mg/L gentamicin; on cetrimide agar plates containing 1 mg/L ciprofloxacin + 8 mg/L gentamicin; on cetrimide agar plates containing 2 mg/L meropenem; and on VRBG agar plates and cetrimide agar plates without antibiotics.

According to the manufacturer’s product information, *P. aeruginosa* shows good growth on VRBG agar plates. The antibiotics combination of 8 mg/L ampicillin + 8 mg/L tetracycline + 2 mg/L gentamicin added to VRBG agar screen plates may inhibit the growth of certain other Gram-negative bacteria and make it feasible to isolate *P. aeruginosa* strains (that have a natural resistance to ampicillin) also from VRBG agar screen plates. Selected isolated colonies were subsequently subcultured on *Pseudomonas*-selective cetrimide agar (VWR International, Leuven, Belgium). The phenotypic characteristics of *P. aeruginosa* strain NL201 included rod-shaped cells under microscopic examination and yellow-green colored colonies on *Pseudomonas* cetrimide agar. An isolated colony was identified by PCR amplification and Sanger sequencing of its 16S rRNA gene (BIOMI Ltd., Gödöllő, Hungary) using the universal primers 27F 5′-AGAGTTTGATCCTGGCTCAG-3′ and 1492R 5′-GGTTACCTTGTTACGACTT-3′ [25]. Its 16S rRNA gene sequence was 100% identical to that of the *P. aeruginosa* reference strain ATCC 10145.

### 2.2. In Vitro Antibiotic Susceptibility Testing of Strain NL201

The in vitro antibiotic susceptibility of *P. aeruginosa* strain NL201 was tested using the disc diffusion method according to EUCAST [26,27,28] using Mueller–Hinton agar plates (OXOID, Basingstoke, UK). In addition, minimal inhibitory concentrations (MICs) of tobramycin and meropenem for *P. aeruginosa* strain NL201 were determined by the broth microdilution method according to EUCAST, and for other isolates against meropenem via the broth microdilution method according to EUCAST [26,27,28].

### 2.3. Whole-Genome Sequencing of P. aeruginosa Strain NL201

*P. aeruginosa* strain NL201 was subjected to whole-genome sequencing (WGS) by iBioScience Ltd. (Pécs, Hungary) on Illumina MiSeq platform (Illumina Inc., San Diego, CA, USA) using 2 × 250 bp paired-end reads. De novo contig-level assembly of the sequencing data was performed using the SPAdes v. 3.15.4 assembler, at 414× genome coverage, and the contig-level draft genome assembly was submitted to the National Center for Biotechnology Information (NCBI) Genomes database under project PRJNA1226900. Further bioinformatic tools available on the Center for Genomic Epidemiology (CGE) platform were applied for a WGS-based characterization of isolate NL201, including the in silico serotyping of *P. aeruginosa* isolates [29], ResFinder [30] and KmerFinder v3.2 [31].

### 2.4. Detection of Acquired Antibiotic Resistance and Virulence Genes

Acquired ARGs were searched for in WGS data using the ABRicate v1.0.1 tool [32,33] against the ResFinder database version 2024-Dec-15 [30,34], with settings of ≥80% threshold for sequence identity [35] and a minimum coverage of ≥80%. *P. aeruginosa* virulence factors were identified against the VFDB database version 2024-Dec-15 [36] at ≥80% coverage and ≥80% identify values. Translated open reading frames (ORFs) were examined using the BLASTP tool against the NCBI Protein database v5. Multivariate clustering of *P. aeruginosa* strains based on their detected virulence factors was carried out using the paired group (UPGMA) algorithm of the PAST 4.08 software (Natural History Museum, University of Oslo, Norway, https://www.nhm.uio.no/english/research/resources/past/, accessed on 31 October 2021).

### 2.5. Searching for Mutations Causing a Quinolone-Resistant Phenotype in P. aeruginosa

Potential amino acid substitutions in the proteins encoded by *gyrA* and *parC* [9,12,13,15] were screened for by alignments with the corresponding translated *gyrA* and *parC* sequences of the *P. aeruginosa* PAO1 reference strain [37,38,39] to detect known amino acid substitutions conferring a quinolone-resistant phenotype in *P. aeruginosa* [9,12,13,15]. The bioinformatic analyses applied in this study were validated for reproducibility [40], where the analyses of the same WGS datasets under codes 3065 and 2064 provided identical results [41].

### 2.6. Construction of Phylogenetic Trees from WGS Data

The reference sequence alignment-based phylogeny builder (REALPHY, Swiss Institute of Bioinformatics, Basel, Switzerland) [42,43] was applied to infer a phylogenetic tree from WGS data of the *P. aeruginosa* strains summarized in Table 1. In the analysis, all WGS sequences were mapped to the selected reference genome of *P. aeruginosa* strain 2875 (Table 1) via bowtie2.

## 3. Results

### 3.1. Isolation of P. aeruginosa Strain NL201 from an Urban Water Drain

The details of the isolation and taxonomic identification of *P. aeruginosa* strain NL201 from a water drain in a subway underpass are described in the Materials and Methods section (see Section 2.1). The 16S rRNA gene sequence of strain NL201 was identical to that of the *P. aeruginosa* reference strain ATCC 10145. WGS and bioinformatic analyses showed that isolate NL201 was a sequence type ST111 serotype O4 *P. aeruginosa* strain. No other *P. aeruginosa* strains were identified during the culturing experiments of the underpass water drain. However, additionally, two *Pseudomonas* sp., two *Morganella* sp. and one *Acinetobacter* sp. isolates were also identified, where a *Pseudomonas* sp. and an *Acinetobacter* sp. isolate were derived using antibiotic-non-containing agar plates.

### 3.2. Antibiotic Susceptibility Testing

The NL201 *P. aeruginosa* strain displayed intermediate resistance to ceftazidime, ciprofloxacin, levofloxacin and meropenem and resistance to piperacillin-tazobactam and tobramycin according to current EUCAST zone diameter breakpoints (Table 2) [26]. The MICs of meropenem and tobramycin for strain NL201 were 4 mg/L and 16 mg/L, respectively, and the associated IC_50_ values were 0.25 mg/L and 3.54 mg/L, respectively. The two *Pseudomonas* sp., two *Morganella* sp. and one *Acinetobacter* sp. isolates obtained from the water drain were tested only for their meropenem MICs that equaled ≤0.5 mg/L. Therefore, these isolates were susceptible to meropenem according to current EUCAST breakpoints for meropenem.

### 3.3. Assessment of ARGs and Known Genetic Mutations Leading to Quinolone Resistance in ST111 P. aeruginosa Strains and WGS Sequenced Isolates from Hungary

**Figure 2:**
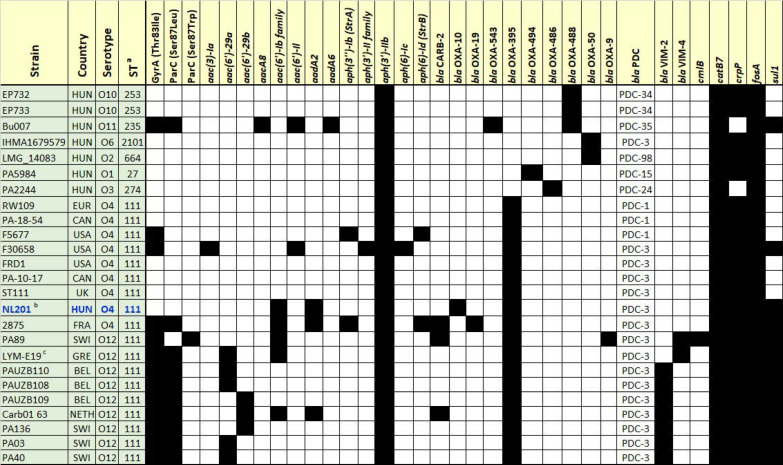
ARGs identified at the minimum coverage of ≥80%, and Thr83Ile and Ser87Leu amino acid substitutions detected in the deduced products of *gyrA* and *parC,* respectively, compared to those in *P. aeruginosa* PAO1, ^a^—ST stands for the sequence type of the isolates as determined by in silico multi-locus sequence typing (MLST) using the Center for Genomic Epidemiology (CGE) platform (https://www.genomicepidemiology.org/). ^b^—The *aac(6′)-Ib* family gene in strain NL201 corresponds to the NG_051697.1 aminoglycoside 6′-N-acetyltransferase gene of the NCBI Bacterial Antimicrobial Resistance Reference Gene Database. ^c^—The *aac(6′)-29a* gene of strain LYM-E19 had a coverage of 67.6% on a contig of 344 nucleotides, and its carriage has been published for strain LYM-E19 [52].

The WGS data of selected *P. aeruginosa* strains listed in Table 1 were analyzed for ARGs and for the presence of known genetic mutations in *gyrA* and *parC* that contribute to quinolone resistance in *P. aeruginosa* isolates [9,12,13,15]. Figure 2 shows that all isolates harbored *catB7*, a chloramphenicol acetyltransferase gene [58]; a class C PDC β-lactamase gene [59]; an OXA-50 type β-lactamase gene [60]; and most of them also carried *crpP*, encoding an enzyme with contradictory results on its potential ciprofloxacin-modifying activity [61]. Furthermore, the analyzed strain set also possessed a number of additional acquired ARGs, including diverse aminoglycoside resistance genes and/or additional β-lactamases or *sul1* (see Figure 2). In addition to the ARGs shown in Figure 2, *P. aeruginosa* strain NL201 also featured a *qacEΔ1* disinfectant resistance determinant.

### 3.4. Identification of Virulence Determinants of ST111 and Other P. aeruginosa Isolates

The *P. aeruginosa* virulence determinants detected against the VFDB reference database are shown in Supplementary Table S1 and Supplementary Figure S1. The total number of identified virulence determinants did not show a significantly different distribution between ST111 and non-ST111 isolates (*p* > 0.05) (Supplementary Table S1). Multivariate clustering of *P. aeruginosa* strains based on their detected virulence factors showed that the environmental NL201 isolate clustered together closely with the ST111 clinical *P. aeruginosa* isolates PA03, PA89 and PA136 characterized recently in Switzerland [50] (Table 1, Supplementary Figure S1). 

### 3.5. Phylogenetic Analysis Based on Whole-Genome Sequencing Data

**Figure 3:**
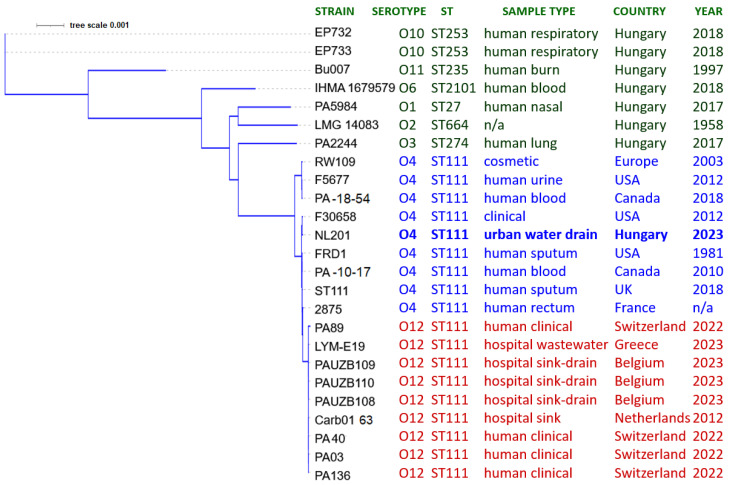
Phylogenetic tree inferred by REALPHY for clinical and environmental *P. aeruginosa* strains. ST numbers provide MLST sequence types. The tree scale is indicated in the upper-left corner. Green, blue and red colors indicate non-ST111, serotype O4 ST111 and serotype O12 ST111 isolates, respectively.

Figure 3 displays the phylogenetic tree inferred by REALPHY using the WGS data of *P. aeruginosa* strains summarized in Table 1. Clustering of the isolates based on their WGS data correlated well with their seven-gene MLST sequence type.

## 4. Discussion

Infections caused by MDR Gram-negative ESKAPE pathogens (*Klebsiella pneumoniae*, *Acinetobacter baumannii*, *P. aeruginosa*, and *Enterobacter* species) are escalating worldwide and represent an increasing public health threat due to their ability to inhabit diverse environments, disseminate via the fecal–oral route, and effectively acquire ARGs [55]. The analysis of natural and human-impacted environments (e.g., aquatic and terrestrial ecosystems, built and urban environments) using culture-based methods and/or metagenomics approaches can provide further insights into the presence, diversity and origins of ARGs and their dissemination. Therefore, such studies can contribute to our understanding of resistance dynamics in and between these diverse environments [41,62,63].

Urban mass transit systems can also serve as a suitable environment for the transmission of bacteria among humans and may act as hotspots for the spread of pathogens and the associated ARGs [64]. Currently, approximately 55% of the world’s population lives in urban areas, and the Metagenomics and Metadesign of the Subways and Urban Biomes (MetaSUB) International Consortium analyzed 4728 metagenomic samples from mass transit systems in 60 cities as part of a worldwide study of the urban microbial ecosystem [65]. Several of the bacterial taxa identified in subways and related urban samples were potentially infectious agents, such as *Staphylococcus*, *Streptococcus*, *Acinetobacter*, *Klebsiella*, and *Enterobacter* species, and among the most prevalent 75 taxa, 5 belonged to the genus *Pseudomonas*: *P. stutzeri*, *P. aeruginosa*, *P. fluorescens*, *P. putida* and *P. balearica* [65]. Consistent with these global findings, several Gram-negative genera potentially associated with human infections were cultured in our study from a mass transit system water drain: *Pseudomonas*, *Acinetobacter* and *Morganella* isolates.

Further analyses of these subway metagenomes with a global distribution revealed that nearly half of the identified ARGs conferred multidrug resistance. The aminoglycoside resistance determinants were the second most abundant, while the β-lactam resistance determinants ranked as the fifth among the most prevalent ARG categories [66].

Infections caused by *P. aeruginosa* in patients suffering from cystic fibrosis usually derive from the environment [63,67]. A study of 12 *P. aeruginosa* strains of soil, water and human origin showed that all strains expressed virulence factors [68]. Virulence determinants among human, bovine, and groundwater *P. aeruginosa* isolates were compared by PCR screening, and the distribution of these virulence determinants was very similar: all tested virulence genes were present in the three major sources, except for *exoU*, which was detected in only one human and one groundwater isolate [68]. Environmental *P. aeruginosa* isolates feature multiple virulence factors and can share the same sequence types (STs) with clinical and epidemic isolates [41,62,63,69,70]. Therefore, it is essential from a One Health perspective to investigate potential urban environmental reservoirs for the major high-risk clones of *P. aeruginosa*. Indeed, based on their global metagenomics study of subways, the MetaSUB consortium concluded that metagenomics approaches alone are insufficient to fully explore the clinical relevance of the bacterial species identified in these urban environments. Therefore, the consortium recommended complementary culture-based studies including the strain-level characterization of virulence factors and antibiotic resistance determinants [65].

Successful MDR clones of bacterial pathogens, also known as high-risk clones, serve as a key way for the dissemination of ARGs [2,71]. In this study, we identified an urban water drain in a heavily populated subway as an environmental source of the global high-risk ST111 *P. aeruginosa* clone. The serotype O4 NL201 isolate carried a set of acquired ARGs, including *aadA2*, *bla*_OXA-10_, *sul1*, and an *aac(6′)-Ib* family aminoglycoside acetyltransferase gene (Figure 2), in addition to generally present intrinsic *P. aeruginosa* resistance genes. Additional characteristics of the NL201 isolate include the presence of the *bla*_PDC-3_ gene, which has been shown to have significantly higher catalytic efficiency against cefepime and imipenem compared to *bla*_PDC-1_ [41,59], as well as the carriage of the potent *P. aeruginosa* virulence factors *exoS, exoT* and *algD* (Supplementary Table S1). Although its OprD porin protein was not disrupted, it contained a range of amino acid substitutions very similar to those found in the PA176 *P. aeruginosa* ST654 human clinical strain characterized in Switzerland [50].

Phylogenetic analysis of a global collection of ST111 *P. aeruginosa* isolates revealed that the chromosomal class C β-lactamase PDC-1 was limited to clade A, while the PDC-3 variant was found in clades B, C1, and C2 [2]. Certain acquired ARGs that clustered within the currently dominant ST111 subclade C2 included *sul1*, *aac(6′)-Ib* family aminoglycoside acetyltransferase genes, *aadA2*, and *bla*_OXA-10_. These ARGs are also harbored by strain NL201. Although nearly all of the dominant subclade C2 isolates had serotype O12 in the analyzed collection of ST111 *P. aeruginosa* (*n* = 969) [2], the serotype O4 strain NL201 characterized in this study has several features that are characteristic for the C2 subclade serotype O12 isolates. These observations suggest that similar recombination and ARG acquisition events associated with the evolution and spread of the ST111 global high-risk *P. aeruginosa* clone may have occurred independently in different geographic locations.

**Figure 4:**
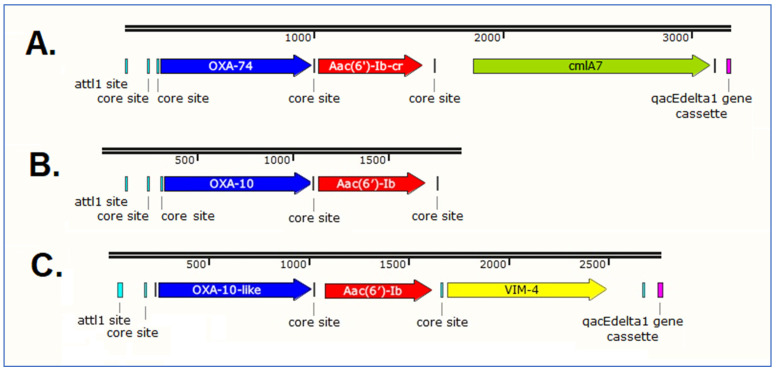
The immediate genetic environment of acquired *bla*_OXA-10_ subgroup and *aac(6′)-Ib-*type ARGs reported in Hungary from *P. aeruginosa* isolates. (**A**) class 1 integron from the PER-1 extended-spectrum β-lactamase producing serotype O11 ST235 clinical *P. aeruginosa* isolate 05-380 from Budapest [6] (**B**) class 1 integron from the serotype O4 ST111 environmental isolate NL201 from Budapest, and (**C**) class 1 integron from the serotype O12 ST229 clinical isolate PA396 from Budapest [5]. Only the 5′-end of the *qacEΔ1* gene cassette is indicated in the figure.

Figure 4 shows a schematic diagram of a class 1 integron from the environmental *P. aeruginosa* strain NL201 in comparison with those from previously characterized *P. aeruginosa* clinical isolates from Budapest. The compared integron structures indicate that a similar arrangement of acquired *bla*_OXA-10_ subgroup and *aac(6′)-Ib-*type ARGs can be observed in an ST111 environmental isolate and also in ST229 and ST235 clinical isolates from the same city (Figure 4). Sequence type ST229 belongs to the ST111 clonal complex [3,4,5]. Since gene cassettes are preferentially inserted into the *attI* site of the class 1 integron [5,72] (Figure 4), these observations suggest that *bla*_OXA-10_ subgroup and *aac(6′)-Ib-*type ARGs could be acquired by integrons circulating within both environmental and clinical bacterial communities. Alternatively, complete integrons of similar gene cassette arrangements may have disseminated via clonal spread/horizontal gene transfer between different high-risk clones.

Serotype O4 isolates of the ST111 global high-risk *P. aeruginosa* clone have now been reported from clinical samples in Canada and the USA, human stool samples in France, and environmental sources (cosmetic product, hospital drain and urban water drain) in various European countries (Table 1, Figure 2 and Figure 3). These findings underscore the effective dissemination of the ST111 global high-risk *P. aeruginosa* clone across different hosts, environments, and habitats and warrant further targeted samplings and investigations of the possible routes of its transmission into human populations.

Based on the molecular analysis of acquired ARGs and the in vitro susceptibility profiles of VIM-producing *P. aeruginosa* isolates reported from Hungary, the presence of a local environmental ARG pool was proposed [5]. Such an environmental reservoir can facilitate interactions between opportunistic pathogens and other environmental bacteria for exchanges of their acquired ARGs and mobile genetic elements outside the clinical settings and under various environmental conditions.

The ARGs detected in the MetaSUB global study showed a “neighborhood” effect within urban samples that were geographically adjacent, where the Jaccard distance increased between sets of ARGs with the geographical distance of their sampling points in the same city [65]. Considering that hospitals are located at approximately 200 m and 500 m distances from the sampling site of *P. aeruginosa* strain NL201 (Figure 1), a similar neighborhood effect may also be assumed between the resistomes of adjacent clinical and urban environmental microbiomes. From this perspective, it is notable that *Pseudomonas* bacteria have also been detected in the air (that is, in the bioaerosol community) of urban public transit samples in different cities [73,74]. The survival of bacterial strains in public transit and subway environments may be further supported by the acquisition of disinfectant resistance genes [75], such as in the case of *P. aeruginosa* strain NL201, which also harbors a *qacEΔ1* disinfectant resistance determinant [76].

The detection of ST111 environmental *P. aeruginosa* isolates in a range of environmental sample types (such as hospital wastewater, hospital sink, urban water drain, see Figure 2) underscores the role of the environment in the dissemination of this high-risk clone. Further characterization of *P. aeruginosa* strain NL201 could involve additional long-read WGS sequencing for a more advanced molecular analysis of its mobile genetic elements, as well as functional assays (such as ExoS/T production, cytotoxicity on cell lines, *Caenorhabditis* elegans model) to demonstrate whether the in silico detected virulence genes are expressed in vivo.

The Agribiotechnology and Precision Breeding for Food Security National Laboratory in Hungary aims to screen for high-risk antibiotic-resistant bacteria in both natural and human-impacted environmental habitats and also in livestock and wildlife. The main objectives of this work are to track the dissemination of key antibiotic-resistant bacterial pathogens and to characterize them using up-to-date culture-based genomics and metagenomics methods. The detection of ST111 *P. aeruginosa* isolates in birds, dogs and ruminant livestock [18,19,20] indeed justifies the validity of a comprehensive One Health approach [77,78] during such targeted future investigations.

## Figures and Tables

**Table 1:** *P. aeruginosa* clinical and environmental strains analyzed in this study ^a^.

Nr.	Strain Code	Location	Sample Type	Year	NCBI Biosample	Ref.
1	Bu007	Hungary	human burn	1997	SAMN04128716	[44]
2	LMG 14083	Hungary	unknown	1958	SAMN04128726	[45]
3	EP732	Hungary	human respiratory	2018	SAMN35303074	[46]
4	EP733	Hungary	human respiratory	2018	SAMN35303075	[46]
5	IHMA 1679579	Hungary	human blood	2018	SAMN24255292	[47]
6	PA5984	Hungary	human nasal	2017	SAMN18653385	[48]
7	PA2244	Hungary	human lung	2017	SAMN18653382	[48]
8	ST111	UK	human sputum	2018	SAMN32301013	[49]
9	PA89	Switzerland	human clinical	2022	SAMN44059281	[50]
10	PA40	Switzerland	human clinical	2022	SAMN44059284	[50]
11	PA03	Switzerland	human clinical	2022	SAMN44059282	[50]
12	PA136	Switzerland	human clinical	2022	SAMN44059266	[50]
13	PAUZB108	Belgium	hospital sink-drain	2023	SAMN41108339	[51]
14	PAUZB109	Belgium	hospital sink-drain	2023	SAMN41108340	[51]
15	PAUZB110	Belgium	hospital sink-drain	2023	SAMN41108341	[51]
16	LYM-E19	Greece	hospital wastewater	2023	SAMN44671623	[52]
17	RW109	Europe	cosmetic	2003	SAMEA104432335	[53]
18	F5677	USA	human urine	2012	SAMN02887043	[54]
19	PA-18-54	Canada	human blood	2018	SAMN36031752	[2]
20	F30658	USA	human clinical	2012	SAMN02894357	[2]
21	NL201	Hungary	water drain	2023	SAMN46966551	this work
22	FRD1	USA	human sputum	1981	SAMN03342417	[2]
23	PA-10-17	Canada	human blood	2010	SAMN36031623	[55]
24	2875	France	human rectum	n.a.	SAMN32874247	[56]
25	Carb01 63	Netherlands	hospital drain	2012	SAMN03389320	[57]

^a^ n.a. stands for not available.

**Table 2:** In vitro antibiotic susceptibility pattern of *P. aeruginosa* strain NL201 ^a^.

Antibiotics Tested								
	CAZ	CZA	TZP	MEM	CIP	LEV	TOB	AK
Inhibitory zone (mm)	25	25	12	14	33	28	12	26
Interpretation	I	S	R	I	I	I	R	S

^a^ Abbreviations for antibiotics: CAZ, ceftazidime; CZA, ceftazidime-avibactam; TZP, piperacillin-tazobactam; MEM, meropenem; CIP, ciprofloxacin; LEV, levofloxacin; TOB, tobramycin; AK, amikacin. Diameters of inhibitory zones are provided in mm, with the following interpretations: S, susceptible; I, intermediate; R, resistant.

## Data Availability

The original contributions presented in this study are included in the article, and further inquiries can be directed to the corresponding author. The contig-level draft genome assembly of *P. aeruginosa* strain NL201 was submitted to the NCBI Genomes database under project PRJNA1226900.

## Supplementary Materials

The following supporting information can be downloaded at: https://www.mdpi.com/article/10.3390/cimb47070532/s1, Table S1: *P. aeruginosa* virulence factors detected in clinical and environmental isolates, Figure S1: Clustering of *P. aeruginosa* isolates based on their virulence factors.
