# Supplementary material for: Detection of the ST111 Global High-Risk Pseudomonas aeruginosa Clone in a Subway Underpass
Source: Curr Issues Mol Biol. 2025 Jul 9;47(7):532. doi: 10.3390/cimb47070532 (PMC12293118; doi:10.3390/cimb47070532)

Supplementary Figure S1. Clustering of *P. aeruginosa* isolates based on their virulence factors

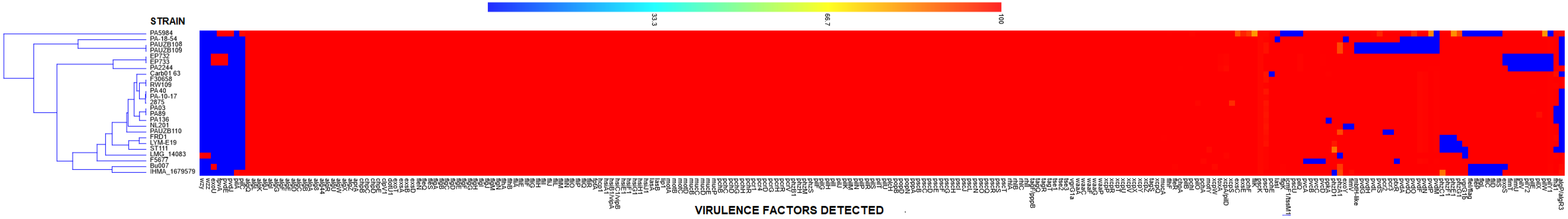

Supplement: Supplementary file 1 [file Supplementary_Figure_S1.pdf › Supplementary_Figure_S1.pdf]
