# Supplementary material for: Detection of the ST111 Global High-Risk Pseudomonas aeruginosa Clone in a Subway Underpass
Source: Curr Issues Mol Biol. 2025 Jul 9;47(7):532. doi: 10.3390/cimb47070532 (PMC12293118; doi:10.3390/cimb47070532)
Supplement: Supplementary file 1 [file Supplementary_Table_S1.pdf › Supplementary_Table_S1.pdf]

Supplementary Table 1. *P. aeruginosa* virulence factors identified with their coverage

Strain	Country	Serotype	ST	NUM_FOUND	*alg44*	*alg8*	*algA*	*algB*	*algC*	*algD*	*algE*	*algF*	*algG*	*algI*	*algJ*	*algK*	*algL*	*algP/algR3*	*algQ*	*algR*	*algU*	*algW*	*algX*	*algZ*	*aprA*	*chpA*	*chpB*	*chpC*	*chpD*	*chpE*	*clpV1*	*dotU1*
2875	FRA	O4	111	231	100	100	100	100	100	100	100	100	100	100	100	100	100	0	100	100	100	100	100	100	100	100	100	100	100	100	100	100
Bu007	HUN	O11	235	226	100	100	100	100	100	100	100	100	100	100	100	100	100	98,87	100	100	100	100	100	100	100	99,9	100	100	100	100	100	100
Carb01 63	NETH	O12	111	230	100	100	100	100	100	100	100	100	100	100	100	100	100	0	100	100	100	100	100	100	100	100	100	100	100	100	100	100
EP732	HUN	O10	253	225	100	100	100	100	100	100	100	100	100	100	100	100	100	0	100	100	100	100	100	100	100	99,9	100	100	100	100	100	100
EP733	HUN	O10	253	225	100	100	100	100	100	100	100	100	100	100	100	100	100	0	100	100	100	100	100	100	100	99,9	100	100	100	100	100	100
F5677	USA	O4	111	227	100	100	100	100	100	100	100	100	100	100	100	100	100	100	100	100	100	100	100	100	100	100	100	100	100	100	100	100
F30658	USA	O4	111	232	100	100	100	100	100	100	100	100	100	100	100	100	100	100	100	100	100	100	100	100	100	100	100	100	100	100	100	100
FRD1	USA	O4	111	228	100	100	100	100	100	100	100	100	100	100	100	100	100	0	100	100	100	100	100	100	100	100	100	100	100	100	100	100
IHMA_1679579	HUN	O6	2101	227	100	100	100	100	100	100	100	100	100	100	100	100	100	97,73	100	100	100	100	100	100	100	100	100	100	100	100	100	100
LMG_14083	HUN	O2	664	232	100	100	100	100	100	100	100	100	100	100	100	100	100	100	100	100	100	100	100	100	100	100	100	100	100	100	100	100
LYM-E19	GRE	O12	111	227	100	100	100	100	100	100	100	100	100	100	100	100	100	0	100	100	100	100	100	100	100	100	100	100	100	100	100	100
NL201	HUN	O4	111	229	100	100	100	100	100	100	100	100	100	100	100	100	100	0	100	100	100	100	100	100	100	100	100	100	100	100	100	100
PA03	SWI	O12	111	230	100	100	100	100	100	100	100	100	100	100	100	100	100	0	100	100	100	100	100	100	100	100	100	100	100	100	100	100
PA40	SWI	O12	111	231	100	100	100	100	100	100	100	100	100	100	100	100	100	0	100	100	100	100	100	100	100	100	100	100	100	100	100	100
PA89	SWI	O12	111	230	100	100	100	100	100	100	100	100	100	100	100	100	100	0	100	100	100	100	100	100	100	100	100	100	100	100	100	100
PA136	SWI	O12	111	229	100	100	100	100	100	100	100	100	100	100	100	100	100	0	100	100	100	100	100	100	100	100	100	100	100	100	100	100
PA-10-17	CAN	O4	111	231	100	100	100	100	100	100	100	100	100	100	100	100	100	0	100	100	100	100	100	100	100	100	100	100	100	100	100	100
PA-18-54	CAN	O4	111	222	100	100	100	100	100	100	100	100	100	100	100	100	100	0	100	100	100	100	100	100	100	100	100	100	100	100	100	100
PA2244	HUN	O3	274	224	100	100	100	100	100	100	100	100	100	100	100	100	100	100	100	100	100	100	100	100	100	99,8	100	100	100	100	100	100
PA5984	HUN	O1	27	223	100	100	100	100	100	100	100	100	100	100	100	100	100	98,58	100	100	100	100	100	100	100	100	100	100	100	100	100	100
PAUZB108	BEL	O12	111	216	100	100	100	100	100	100	100	100	100	100	100	100	100	0	100	100	100	100	100	100	100	100	100	100	100	100	100	100
PAUZB109	BEL	O12	111	216	100	100	100	100	100	100	100	100	100	100	100	100	100	0	100	100	100	100	100	100	100	100	100	100	100	100	100	100
PAUZB110	BEL	O12	111	229	100	100	100	100	100	100	100	100	100	100	100	100	100	0	100	100	100	100	100	100	100	100	100	100	100	100	100	100
RW109	EUR	O4	111	232	100	100	100	100	100	100	100	100	100	100	100	100	100	100	100	100	100	100	100	100	100	100	100	100	100	100	100	100
ST111	UK	O4	111	225	100	100	100	100	100	100	100	100	100	100	100	100	100	0	100	100	100	100	100	100	100	100	100	100	100	100	100	100

Strain	*exoS*	*exoT*	*exoU*	*exoY*	*exsA*	*exsB*	*exsC*	*exsD*	*exsE*	*fha1*	*fimT*	*fimU*	*fimV*	*fleI/flag*	*fleN*	*fleP*	*fleQ*	*fleR*	*fleS*
2875	100	100	0	100	100	100	100	100	100	100	100	100	100	100	100	100	100	100	100
Bu007	0	100	100	99,9	100	100	100	100	100	100	100	100	100	0	100	0	100	99,6	100
Carb01 63	100	100	0	100	100	100	100	100	100	98	100	100	100	100	100	100	100	100	100
EP732	0	100	100	99,9	100	100	100	100	100	100	0	0	100	100	100	100	100	100	100
EP733	0	100	100	99,9	100	100	100	100	100	100	0	0	100	100	100	100	100	100	100
F5677	100	100	0	100	100	100	100	100	100	100	100	100	100	100	100	100	100	100	100
F30658	100	100	0	99,9	100	100	100	100	100	100	100	100	100	100	100	100	100	100	100
FRD1	100	100	0	100	100	100	100	100	100	99,2	100	100	100	100	100	100	100	100	100
IHMA_1679579	100	100	0	100	100	100	100	100	100	100	100	100	98,4	0	100	0	100	100	100
LMG_14083	100	100	0	100	100	100	100	100	100	99,6	100	100	100	100	100	100	100	100	100
LYM-E19	100	100	0	100	100	100	100	100	100	100	100	100	100	100	100	100	100	100	100
NL201	100	100	0	0	100	100	100	100	100	100	100	100	0	100	100	100	100	100	100
PA03	100	100	0	100	100	100	100	100	100	0	100	100	100	100	100	100	100	100	100
PA40	100	100	0	99,9	100	100	100	100	100	100	100	100	100	100	100	100	100	100	100
PA89	100	100	0	100	100	100	100	100	100	0	100	100	100	100	100	100	100	100	100
PA136	100	100	0	100	100	100	100	100	100	0	100	100	100	100	100	100	100	100	100
PA-10-17	100	100	0	100	100	100	100	100	100	100	100	100	100	100	100	100	100	100	100
PA-18-54	100	100	0	0	100	100	100	100	100	100	100	100	100	100	100	100	100	100	100
PA2244	100	100	0	100	100	100	100	100	100	100	0	0	100	100	100	100	100	100	100
PA5984	100	100	0	100	100	100	89	100	95,5	99,6	100	100	100	0	100	0	100	100	100
PAUZB108	100	100	0	100	100	100	100	100	100	100	100	100	100	100	100	100	100	100	100
PAUZB109	100	100	0	100	100	100	100	100	100	100	100	100	100	100	100	100	100	100	100
PAUZB110	100	100	0	100	100	100	100	100	100	100	100	100	100	100	100	100	100	100	100
RW109	100	100	0	100	100	100	100	100	100	99,6	100	100	100	100	100	100	100	100	100
ST111	100	100	0	100	100	100	100	100	100	0	100	100	100	100	100	100	100	100	100

Strain	*flgA*	*flgB*	*flgC*	*flgD*	*flgE*	*flgF*	*flgG*	*flgH*	*flgI*	*flgJ*	*flgK*	*flgL*	*flgM*	*flgN*	*flhA*	*flhB*	*flhF*	*fliA*	*fliC*	*fliD*	*fliE*	*fliF*	*fliG*	*fliH*	*fliI*	*fliJ*	*fliK*	*fliL*	*fliM*	*fliN*	*fliO*
2875	100	100	100	100	100	100	100	100	100	100	100	100	100	100	100	100	100	100	100	100	100	100	100	100	100	100	100	100	100	100	100
Bu007	100	100	100	100	100	100	100	100	100	100	99,7	0	100	100	100	100	100	100	0	0	100	100	100	100	100	100	100	100	100	100	100
Carb01 63	100	100	100	100	100	100	100	100	100	100	100	100	100	100	100	100	100	100	100	100	100	100	100	100	100	100	100	100	100	100	100
EP732	100	100	100	100	100	100	100	100	100	100	100	100	100	100	100	100	100	100	100	100	100	100	100	100	100	100	100	100	100	100	100
EP733	100	100	100	100	100	100	100	100	100	100	100	100	100	100	100	100	100	100	100	100	100	100	100	100	100	100	100	100	100	100	100
F5677	100	100	100	100	100	100	100	100	100	100	100	100	100	100	100	100	100	100	100	100	100	100	100	100	100	100	100	100	100	100	100
F30658	100	100	100	100	100	100	100	100	100	100	100	100	100	100	100	100	100	100	100	100	100	100	100	100	100	100	100	100	100	100	100
FRD1	100	100	100	100	100	100	100	100	100	100	100	100	100	100	100	100	100	100	100	100	100	100	100	100	100	100	100	100	100	100	100
IHMA_1679579	100	100	100	100	100	100	100	100	100	100	99,7	0	100	100	100	100	100	100	0	0	100	100	100	100	100	100	100	100	100	100	100
LMG_14083	100	100	100	100	100	100	100	100	100	100	100	100	100	100	100	100	100	100	100	100	100	100	100	100	100	100	100	100	100	100	100
LYM-E19	100	100	100	100	100	100	100	100	100	100	100	100	100	100	100	100	100	100	100	100	100	100	100	100	100	100	100	100	100	100	100
NL201	100	100	100	100	100	100	100	100	100	100	100	100	100	100	100	100	100	100	100	100	100	100	100	100	100	100	100	100	100	100	100
PA03	100	100	100	100	100	100	100	100	100	100	100	100	100	100	100	100	100	100	100	100	100	100	100	100	100	100	100	100	100	100	100
PA40	100	100	100	100	100	100	100	100	100	100	100	100	100	100	100	100	100	100	100	100	100	100	100	100	100	100	100	100	100	100	100
PA89	100	100	100	100	100	100	100	100	100	100	100	100	100	100	100	100	100	100	100	100	100	100	100	100	100	100	100	100	100	100	100
PA136	100	100	100	100	100	100	100	100	100	100	100	100	100	100	100	100	100	100	100	100	100	100	100	100	100	100	100	100	100	100	100
PA-10-17	100	100	100	100	100	100	100	100	100	100	100	100	100	100	100	100	100	100	100	100	100	100	100	100	100	100	100	100	100	100	100
PA-18-54	100	100	100	100	100	100	100	100	100	100	100	100	100	100	100	100	100	100	100	100	100	100	100	100	100	100	100	100	100	100	100
PA2244	100	100	100	100	100	100	100	100	100	100	100	100	100	100	100	100	100	100	100	100	100	100	100	100	100	100	100	100	100	100	100
PA5984	100	100	100	100	100	100	100	100	100	100	0	0	100	100	100	100	99,8	100	0	0	100	100	100	100	100	100	80,1	100	100	100	100
PAUZB108	100	100	100	100	100	100	100	100	100	100	100	100	100	100	100	100	100	100	100	100	100	100	100	100	100	100	100	100	100	100	100
PAUZB109	100	100	100	100	100	100	100	100	100	100	100	100	100	100	100	100	100	100	100	100	100	100	100	100	100	100	100	100	100	100	100
PAUZB110	100	100	100	100	100	100	100	100	100	100	100	100	100	100	100	100	100	100	100	100	100	100	100	100	100	100	100	100	100	100	100
RW109	100	100	100	100	100	100	100	100	100	100	100	100	100	100	100	100	100	100	100	100	100	100	100	100	100	100	100	100	100	100	100
ST111	100	100	100	100	100	100	100	100	100	100	100	100	100	100	100	100	100	100	100	100	100	100	100	100	100	100	100	100	100	100	100

Strain	*fliP*	*fliQ*	*fliR*	*fliS*	*fptA*	*fpvA*	*hcp1*	*hsiA1*	*hsiB1/vipA*	*hsiC1/vipB*	*hsiE1*	*hsiF1*	*hsiG1*	*hsiH1*	*hsiJ1*	*icmF1/tssM1*	*lasA*	*lasB*	*lasI*	*lip1*	*mbtH-like*	*motA*	*motB*	*motC*	*motD*	*motY*
2875	100	100	100	100	100	0	100	100	100	100	100	100	100	100	100	100	100	100	100	100	100	100	100	100	100	100
Bu007	100	100	100	0	100	0	100	100	100	100	100	100	100	100	100	100	100	100	100	100	100	100	100	100	100	100
Carb01 63	100	100	100	100	100	0	100	100	100	100	100	100	100	100	100	100	100	100	100	100	100	100	100	100	100	100
EP732	100	100	100	100	100	100	100	100	100	100	100	100	100	100	100	100	100	100	100	100	100	100	100	100	100	100
EP733	100	100	100	100	100	100	100	100	100	100	100	100	100	100	100	100	100	100	100	100	100	100	100	100	100	100
F5677	100	100	100	100	100	0	100	100	100	100	100	100	100	100	100	100	100	100	100	100	100	100	100	100	100	100
F30658	100	100	100	100	100	0	100	100	100	100	100	100	100	100	100	100	100	100	100	100	100	100	100	100	100	100
FRD1	100	100	100	100	100	0	100	100	100	100	100	100	100	100	100	100	100	100	100	100	100	100	100	100	100	100
IHMA_1679579	100	100	100	0	100	0	100	100	100	100	100	100	100	100	100	100	100	100	100	100	100	100	100	100	100	100
LMG_14083	100	100	100	100	100	0	100	100	100	100	100	100	100	100	100	100	100	100	100	100	100	100	100	100	100	100
LYM-E19	100	100	100	100	100	0	100	100	100	100	100	100	100	100	100	100	100	100	100	100	100	100	100	100	100	100
NL201	100	100	100	100	100	0	100	100	100	100	100	100	100	100	100	100	100	100	100	100	100	100	100	100	100	100
PA03	100	100	100	100	100	0	100	100	100	100	100	100	100	100	100	100	100	100	100	100	100	100	100	100	100	100
PA40	100	100	100	100	100	0	100	100	100	100	100	100	100	100	100	100	100	100	100	100	100	100	100	100	100	100
PA89	100	100	100	100	100	0	100	100	100	100	100	100	100	100	100	100	100	100	100	100	100	100	100	100	100	100
PA136	100	100	100	100	100	0	100	100	100	100	100	100	100	100	100	100	100	100	100	100	100	100	100	100	100	100
PA-10-17	100	100	100	100	100	0	100	100	100	100	100	100	100	100	100	100	100	100	100	100	100	100	100	100	100	100
PA-18-54	100	100	100	100	100	0	100	100	100	100	100	100	100	100	100	100	100	100	0	100	100	100	100	100	100	100
PA2244	100	100	100	100	100	0	100	100	100	100	100	100	100	100	100	100	100	100	100	100	100	100	100	100	100	100
PA5984	100	100	100	0	100	100	100	100	100	100	100	100	100	100	100	0	100	100	100	100	100	100	100	100	100	100
PAUZB108	100	100	100	100	100	0	100	100	100	100	100	100	100	100	100	100	100	100	100	100	0	100	100	100	100	100
PAUZB109	100	100	100	100	100	0	100	100	100	100	100	100	100	100	100	100	100	100	100	100	0	100	100	100	100	100
PAUZB110	100	100	100	100	100	0	100	100	100	100	100	100	100	100	100	100	100	100	100	100	100	100	100	100	100	100
RW109	100	100	100	100	100	0	100	100	100	100	100	100	100	100	100	100	100	100	100	100	100	100	100	100	100	100
ST111	100	100	100	100	100	0	100	100	100	100	100	100	100	100	100	100	100	100	100	100	100	100	100	100	100	98,8

Strain	*mucA*	*mucB*	*mucC*	*mucD*	*mucE*	*mucP*	*pchA*	*pchB*	*pchC*	*pchD*	*pchE*	*pchF*	*pchG*	*pchH*	*pchI*	*pchR*	*pcr1*	*pcr2*	*pcr3*	*pcr4*	*pcrD*	*pcrG*	*pcrH*	*pcrR*	*pcrV*
2875	100	100	100	100	100	100	100	100	100	100	100	100	100	100	100	100	100	100	100	100	100	100	100	100	100
Bu007	100	100	100	100	100	100	100	100	100	100	100	100	100	100	100	100	100	100	100	100	100	100	100	100	100
Carb01 63	100	100	100	100	100	100	100	100	100	100	0	100	100	100	100	100	100	100	100	100	100	100	100	100	100
EP732	100	100	100	100	100	100	100	100	100	100	100	100	100	100	100	100	100	100	100	100	100	100	100	100	100
EP733	100	100	100	100	100	100	100	100	100	100	100	100	100	100	100	100	100	100	100	100	100	100	100	100	100
F5677	100	100	100	100	100	100	99,1	100	100	100	100	100	100	100	100	100	100	100	100	100	100	100	100	100	100
F30658	100	100	100	100	100	100	100	100	100	100	100	100	100	100	100	100	100	100	100	100	100	100	100	100	100
FRD1	99,8	100	100	100	100	100	100	100	100	100	100	100	100	100	100	100	100	100	100	100	100	100	100	100	100
IHMA_1679579	100	100	100	100	100	100	100	100	100	100	100	100	100	100	100	100	100	100	100	100	100	100	100	100	100
LMG_14083	100	100	100	100	100	100	100	100	100	100	100	100	100	100	100	100	100	100	100	100	100	100	100	100	100
LYM-E19	100	100	100	100	100	100	100	100	100	100	100	100	100	100	100	100	100	100	100	100	100	100	100	100	100
NL201	100	100	100	100	100	100	100	100	100	100	100	100	100	100	100	100	100	100	100	100	100	100	100	100	100
PA03	100	100	100	100	100	100	100	100	100	100	100	100	100	100	100	100	100	100	100	100	100	100	100	100	100
PA40	100	100	100	100	100	100	100	100	100	100	100	100	100	100	100	100	100	100	100	100	100	100	100	100	100
PA89	100	100	100	100	100	100	100	100	100	100	100	100	100	100	100	100	100	100	100	100	100	100	100	100	100
PA136	100	100	100	100	100	100	100	100	100	100	100	100	100	100	100	100	100	100	100	100	100	100	100	100	100
PA-10-17	100	100	100	100	100	100	100	100	100	100	100	100	100	100	100	100	100	100	100	100	100	100	100	100	100
PA-18-54	100	100	100	100	100	100	100	100	100	100	100	99,9	100	100	100	100	100	100	100	100	100	100	100	100	100
PA2244	100	100	100	100	100	100	100	100	100	100	100	100	100	100	99,9	100	100	100	100	100	100	100	100	100	100
PA5984	100	100	100	100	100	100	100	100	100	100	100	92,5	100	100	99,4	100	100	100	100	100	100	100	100	100	100
PAUZB108	100	100	100	100	100	100	100	100	100	100	100	100	100	100	100	100	100	0	0	100	100	100	100	100	100
PAUZB109	100	100	100	100	100	100	100	100	100	100	100	100	100	100	100	100	100	0	0	100	100	100	100	100	100
PAUZB110	100	100	100	100	100	100	100	100	100	100	100	100	100	100	100	100	100	0	0	100	100	100	100	100	100
RW109	100	100	100	100	100	100	100	100	100	100	100	100	100	100	100	100	100	100	100	100	100	100	100	100	100
ST111	100	100	100	100	100	100	100	100	100	100	100	100	100	100	100	100	100	100	100	100	100	100	100	100	100

Strain	*phzA1*	*phzB1*	*phzC1*	*phzD1*	*phzE1*	*phzF1*	*phzG1*	*phzH*	*phzM*	*phzS*	*pilA*	*pilB*	*pilC*	*pilE*	*pilF*	*pilG*	*pilH*	*pilI*	*pilJ*	*pilK*	*pilM*	*pilN*	*pilO*	*pilP*	*pilQ*	*pilR*	*pilS*	*pilT*	*pilU*	*pilV*	*pilW*	*pilX*	*pilY1*	*pilY2*
2875	100	100	100	100	100	100	100	100	100	100	0	100	0	100	100	100	100	100	100	100	100	100	99,2	100	100	100	100	100	100	100	100	100	99,7	100
Bu007	92,4	100	100	100	100	100	100	100	100	100	0	100	0	100	100	100	100	100	100	100	100	100	100	100	100	100	100	100	100	100	100	100	99,6	100
Carb01 63	100	100	100	100	100	100	100	100	100	100	0	100	0	100	100	100	100	100	100	100	100	100	100	100	100	100	100	100	100	100	100	100	99,7	100
EP732	100	100	100	100	100	100	100	100	100	100	0	99,9	0	0	100	100	100	100	100	100	100	100	100	100	99,4	100	100	100	100	0	0	0	0	0
EP733	100	100	100	100	100	100	100	100	100	100	0	99,9	0	0	100	100	100	100	100	100	100	100	100	100	99,4	100	100	100	100	0	0	0	0	0
F5677	100	100	100	100	100	100	100	100	100	100	0	100	0	100	100	100	100	100	100	100	100	100	100	100	100	100	100	100	100	100	100	100	99,7	100
F30658	100	100	100	100	100	100	100	100	100	100	0	100	0	100	100	100	100	100	100	100	100	100	100	100	100	100	100	100	100	100	100	100	99,7	100
FRD1	100	100	0	100	0	0	100	100	100	100	0	100	0	100	100	100	100	100	100	100	100	100	100	100	100	100	100	100	100	100	100	100	99,7	100
IHMA_1679579	90,6	100	84,9	0	100	100	100	100	100	100	98	100	100	100	100	100	100	100	100	100	100	100	100	100	100	100	100	100	100	100	100	100	99,4	100
LMG_14083	0	100	100	100	100	100	100	100	100	100	0	100	0	100	100	100	100	100	100	100	100	100	100	100	100	100	100	100	100	100	100	100	99,7	100
LYM-E19	100	100	0	97	0	0	0	100	100	100	0	100	0	100	100	100	100	100	100	100	100	100	100	100	100	100	100	100	100	100	100	100	99,7	100
NL201	100	100	100	100	100	100	100	100	100	100	0	100	0	100	100	100	100	100	100	100	100	100	100	100	100	100	100	100	100	100	100	100	99,7	100
PA03	100	100	100	100	100	100	100	100	100	100	0	100	0	100	100	100	100	100	100	100	100	100	100	100	100	100	100	100	100	100	100	100	99,7	100
PA40	100	100	100	100	100	100	100	100	100	100	0	100	0	99,8	100	100	100	100	100	100	100	100	100	100	100	100	100	100	100	100	100	100	99,7	100
PA89	100	100	100	100	100	100	100	100	100	100	0	100	0	100	100	100	100	100	100	100	100	100	100	100	100	100	100	100	100	100	100	98	99,7	100
PA136	100	100	100	100	100	100	100	100	100	100	0	100	0	100	100	100	100	100	100	100	100	100	100	100	100	100	100	100	100	100	100	100	99,7	100
PA-10-17	100	100	100	100	100	100	100	100	100	100	0	100	0	100	100	100	100	100	100	100	100	100	100	100	100	100	100	100	100	100	100	100	99,7	100
PA-18-54	100	100	100	100	100	100	100	100	100	100	0	100	0	100	100	100	100	100	100	100	100	100	100	100	100	100	100	100	100	100	100	100	99,7	100
PA2244	100	100	100	100	100	100	100	100	100	100	0	99,9	0	0	100	100	100	100	100	100	100	100	100	100	99,4	100	100	100	100	0	0	0	0	0
PA5984	100	100	100	100	81,4	100	90,5	100	100	100	0	100	98,7	100	100	100	100	100	100	100	100	100	100	100	0	100	100	100	100	100	80,1	100	0	100
PAUZB108	90,6	100	100	100	100	100	100	100	100	100	0	100	0	100	100	100	100	100	100	100	100	100	100	100	100	100	100	100	100	100	100	100	99,7	100
PAUZB109	90,6	100	100	100	100	100	100	100	100	100	0	100	0	100	100	100	100	100	100	100	100	100	100	100	100	100	100	100	100	100	100	100	99,7	100
PAUZB110	90,6	100	100	100	100	100	100	100	100	100	0	100	0	100	100	100	100	100	100	100	100	100	100	100	100	100	100	100	100	100	100	100	99,7	100
RW109	100	100	100	100	100	100	100	100	100	100	0	100	0	100	100	100	100	100	100	100	100	100	100	100	100	100	100	100	100	100	100	100	99,7	100
ST111	100	100	0	84,5	0	0	0	100	100	100	0	100	0	100	100	100	100	100	100	100	100	100	100	100	100	100	100	100	100	100	100	100	99,7	100

Strain	*plcH*	*popB*	*popD*	*popN*	*ppkA*	*pppA*	*pscB*	*pscC*	*pscD*	*pscE*	*pscF*	*pscG*	*pscH*	*pscI*	*pscJ*	*pscK*	*pscL*	*pscN*	*pscO*	*pscP*	*pscQ*	*pscR*	*pscS*	*pscT*	*pscU*
2875	100	100	100	100	100	100	100	100	100	100	100	100	100	100	100	99	100	100	100	97,3	100	100	100	100	100
Bu007	100	100	100	100	100	100	100	100	100	100	100	100	100	100	100	99	100	100	100	99,5	100	100	100	100	100
Carb01 63	100	100	100	100	99,7	100	100	100	100	100	100	100	100	100	100	99	100	100	100	97,3	100	100	100	100	100
EP732	100	100	100	100	100	100	100	100	100	100	100	100	100	100	100	99	100	100	100	100	100	100	100	100	100
EP733	100	100	100	100	100	100	100	100	100	100	100	100	100	100	100	99	100	100	100	100	100	100	100	100	100
F5677	100	100	100	100	100	100	100	100	100	100	100	100	100	100	100	99	100	100	100	99,5	100	100	100	100	100
F30658	100	100	100	100	100	100	100	100	100	100	100	100	100	100	100	99	100	100	100	97,8	100	100	100	100	100
FRD1	100	100	100	100	100	100	100	100	100	100	100	100	100	100	100	99	100	100	100	98,4	100	100	100	100	100
IHMA_1679579	100	100	100	100	100	100	100	100	100	100	100	100	100	100	100	99	100	100	100	100	100	100	100	100	100
LMG_14083	100	100	100	100	100	100	100	100	100	100	100	100	100	100	100	99	100	100	100	99,5	100	100	100	100	100
LYM-E19	100	100	100	100	100	100	100	100	100	100	100	100	100	100	100	99	100	100	100	97,8	100	100	100	100	100
NL201	100	100	100	100	100	100	100	100	100	100	100	100	100	100	100	99	100	100	100	97,3	100	100	100	100	100
PA03	100	100	100	100	100	100	100	100	100	100	100	100	100	100	100	99	100	100	100	97,8	100	100	100	100	100
PA40	100	100	100	100	100	100	100	100	100	100	100	100	100	100	100	99	100	100	100	97,3	100	100	100	100	100
PA89	100	100	100	100	100	100	100	100	100	100	100	100	100	100	100	99	100	100	100	97,3	100	100	100	100	100
PA136	100	100	100	100	0	100	100	100	100	100	100	100	100	100	100	99	100	100	100	97,8	100	100	100	100	100
PA-10-17	100	100	100	100	100	100	100	100	100	100	100	100	100	100	100	99	100	100	100	98,9	100	100	100	100	100
PA-18-54	100	100	100	100	100	100	100	100	100	100	100	100	100	100	100	99	100	100	100	99,5	100	100	100	100	100
PA2244	100	100	100	100	100	100	100	100	100	100	100	100	100	100	100	99	100	100	100	100	100	100	100	100	100
PA5984	100	100	100	100	100	100	100	100	100	100	100	100	100	100	100	99	100	100	100	98,9	100	100	100	100	0
PAUZB108	100	100	100	100	100	100	100	100	100	100	100	100	100	100	100	99	100	100	100	97,8	100	100	100	100	100
PAUZB109	100	100	100	100	100	100	100	100	100	100	100	100	100	100	100	99	100	100	100	97,8	100	100	100	100	100
PAUZB110	100	100	100	100	100	100	100	100	100	100	100	100	100	100	100	99	100	100	100	97,8	100	100	100	100	100
RW109	100	100	100	100	100	100	100	100	100	100	100	100	100	100	100	99	100	100	100	99,5	100	100	100	100	100
ST111	100	100	100	100	100	100	100	100	100	100	100	100	100	100	100	99	100	100	100	99,5	100	100	100	100	100

Strain	*ptxR*	*pvcA*	*pvcB*	*pvcC*	*pvcD*	*pvdA*	*pvdE*	*pvdF*	*pvdG*	*pvdH*	*pvdJ*	*pvdL*	*pvdM*	*pvdN*	*pvdO*	*pvdP*	*pvdQ*	*pvdS*	*rhlA*	*rhlB*	*rhlC*	*rhlI*
2875	100	100	100	100	100	100	0	99,2	100	100	0	100	99,7	99,5	100	100	100	100	100	100	100	100
Bu007	100	100	100	100	99,9	100	0	100	100	100	0	100	99,8	100	100	99,8	100	100	100	100	100	100
Carb01 63	100	100	100	100	100	100	0	99,2	100	100	0	100	99,7	99,5	100	100	100	100	100	100	100	100
EP732	100	100	100	100	99,9	100	100	100	100	100	0	100	100	100	100	100	100	100	100	100	100	100
EP733	100	100	100	100	99,9	100	100	100	100	100	0	100	100	100	100	100	100	100	100	100	100	100
F5677	0	0	0	0	0	100	0	99,2	100	100	0	100	99,7	99,5	100	100	100	100	100	100	100	100
F30658	100	100	100	100	100	100	0	99,2	100	100	0	100	99,7	99,5	100	100	100	100	100	100	100	100
FRD1	100	100	100	100	100	100	0	99,2	100	100	0	100	99,7	99,5	100	100	100	100	100	100	100	100
IHMA_1679579	100	100	100	100	100	100	0	100	100	100	0	100	99,8	100	100	99,8	100	100	100	100	100	100
LMG_14083	100	100	100	100	100	100	0	100	100	100	0	100	100	100	100	100	100	100	100	100	100	100
LYM-E19	100	100	100	100	100	100	0	99,2	100	100	0	100	99,7	99,5	100	100	100	100	100	100	100	100
NL201	100	100	100	100	100	100	0	99,2	100	100	0	100	99,7	99,5	100	100	100	100	100	100	100	100
PA03	100	100	100	100	100	100	0	99,2	100	100	0	100	99,7	99,5	100	100	100	100	100	100	100	100
PA40	100	100	100	100	100	100	0	99,2	100	100	0	100	99,7	99,5	100	100	100	100	100	100	100	100
PA89	100	100	100	100	100	100	0	99,2	100	100	0	100	99,7	99,5	100	100	100	100	100	100	100	100
PA136	100	100	100	100	100	100	0	99,2	100	100	0	100	99,7	99,5	100	100	100	100	100	100	100	100
PA-10-17	100	100	100	100	100	100	0	99,2	100	100	0	100	99,7	99,5	100	100	100	100	100	100	100	100
PA-18-54	100	100	100	100	100	0	0	0	100	100	0	100	0	0	0	0	0	100	100	100	100	100
PA2244	100	100	100	100	100	100	0	100	100	100	0	100	99,8	100	100	99,8	100	100	100	100	100	100
PA5984	100	100	100	100	100	100	100	100	100	100	100	99,5	0	100	93,6	82	100	95	100	100	100	100
PAUZB108	0	100	100	100	100	0	0	0	0	0	0	0	0	0	0	0	0	0	100	100	100	100
PAUZB109	0	100	100	100	100	0	0	0	0	0	0	0	0	0	0	0	0	0	100	100	100	100
PAUZB110	100	100	100	100	100	100	0	99,2	100	100	0	100	99,7	99,5	100	100	100	100	100	100	100	100
RW109	100	100	100	100	100	100	0	99,2	100	100	0	100	99,7	99,5	100	100	100	100	100	100	100	100
ST111	100	100	100	100	100	100	0	99,2	100	100	0	100	99,7	99,5	100	100	100	100	100	100	100	100

Strain	*tagF/pppB*	*tagQ*	*tagR*	*tagS*	*tagT*	*toxA*	*tse1*	*tse2*	*tse3*	*vgrG1a*	*vgrG1b*	*waaA*	*waaC*	*waaF*	*waaG*	*waaP*	*wzy*	*wzz*	*xcpA/pilD*	*xcpP*	*xcpQ*	*xcpR*	*xcpS*	*xcpT*	*xcpU*	*xcpV*	*xcpW*	*xcpX*	*xcpY*	*xcpZ*
2875	100	100	100	100	100	100	100	100	100	100	100	100	100	100	100	100	0	0	100	100	100	100	94,2	100	100	100	100	100	100	100
Bu007	100	100	100	100	100	100	100	100	100	100	100	100	100	100	100	100	0	0	100	100	100	100	100	100	100	100	98,7	100	100	100
Carb01 63	100	100	100	100	100	100	100	100	100	100	100	100	100	100	100	100	0	0	100	100	100	100	100	100	100	100	100	100	100	100
EP732	100	100	100	100	100	99,5	100	100	100	100	100	100	100	100	100	100	0	0	99,3	100	100	100	100	100	100	100	100	100	100	100
EP733	100	100	100	100	100	99,5	100	100	100	100	100	100	100	100	100	100	0	0	99,3	100	100	100	100	100	100	100	100	100	100	100
F5677	100	100	100	100	100	100	100	100	100	100	100	100	100	100	100	100	0	0	100	100	100	100	100	100	100	100	100	100	100	100
F30658	100	100	100	100	100	100	100	100	100	100	100	100	100	100	100	100	0	0	100	100	100	100	100	100	100	100	100	100	100	100
FRD1	100	100	100	100	100	100	100	100	100	100	100	100	100	100	100	100	0	0	100	100	100	100	100	100	100	100	100	100	100	100
IHMA_1679579	100	100	100	100	100	100	100	100	100	100	100	100	100	100	100	100	0	0	100	100	99,9	100	100	100	100	100	100	100	100	100
LMG_14083	100	100	100	100	100	100	100	100	100	100	0	100	100	100	100	100	100	100	99,3	100	100	100	100	100	100	100	100	100	100	100
LYM-E19	100	100	100	100	100	100	100	100	100	100	100	100	100	100	100	100	0	0	100	100	100	100	100	100	100	100	100	100	100	100
NL201	100	100	100	100	100	100	100	100	100	100	100	100	100	100	100	100	0	0	100	100	100	100	100	100	100	100	100	100	100	100
PA03	100	100	100	100	100	100	100	100	100	100	100	100	100	100	100	100	0	0	100	100	100	100	100	100	100	100	100	100	100	100
PA40	100	100	100	100	100	100	100	100	100	100	100	100	100	100	100	100	0	0	100	100	100	100	100	100	100	100	100	100	100	100
PA89	100	100	100	100	100	100	100	100	100	100	100	100	100	100	100	100	0	0	100	100	100	100	100	100	100	100	100	100	100	100
PA136	100	100	100	100	100	100	100	100	100	100	100	100	100	100	100	100	0	0	100	100	100	100	100	100	100	100	100	100	100	100
PA-10-17	100	100	100	100	100	100	100	100	100	100	100	100	100	100	100	100	0	0	100	100	100	100	100	100	100	100	100	100	100	100
PA-18-54	100	100	100	99,9	100	100	100	100	100	100	100	100	100	100	100	100	0	0	100	100	100	100	100	100	100	100	100	100	100	100
PA2244	100	100	100	100	100	99,2	100	100	100	100	100	100	100	100	100	100	0	0	98,6	100	100	100	100	100	100	100	100	100	100	100
PA5984	100	100	100	100	100	100	100	100	100	100	0	100	100	100	100	100	0	0	100	100	100	100	100	100	100	100	100	100	100	100
PAUZB108	100	100	100	100	100	100	100	100	100	100	100	100	100	100	100	100	0	0	100	100	100	100	100	100	100	100	100	100	100	100
PAUZB109	100	100	100	100	100	100	100	100	100	100	100	100	100	100	100	100	0	0	100	100	100	100	100	100	100	100	100	100	100	100
PAUZB110	100	100	100	100	100	100	100	100	100	100	100	100	100	100	100	100	0	0	100	100	100	100	100	100	100	100	100	100	100	100
RW109	100	100	100	100	100	100	100	100	100	100	100	100	100	100	100	100	0	0	100	100	100	100	100	100	100	100	100	100	100	100
ST111	100	100	100	100	100	100	100	100	100	100	0	100	100	100	100	100	0	0	100	100	100	100	100	100	100	100	100	100	100	100
